# Clinical Characteristics of Immune Response in Asymptomatic Carriers and Symptomatic Patients With COVID-19

**DOI:** 10.3389/fmicb.2022.896965

**Published:** 2022-05-24

**Authors:** Entao Li, Shen Wang, Wenwen He, Jun He, Luogeng Liu, Xiaotuan Zhang, Songtao Yang, Feihu Yan, Yuwei Gao, Bin Liu, Xianzhu Xia

**Affiliations:** ^1^Key Laboratory of Jilin Province for Zoonosis Prevention and Control, Changchun Veterinary Research Institute, Chinese Academy of Agricultural Sciences, Changchun, China; ^2^Department of Laboratory Medicine, The Second Affiliated Hospital, University of South China, Hengyang, China; ^3^Department of Laboratory Medicine, Nanhua Hospital, University of South China, Hengyang, China

**Keywords:** COVID-19, SARS-CoV-2, asymptomatic carriers, serology, cytokine, antibody

## Abstract

The pandemic of coronavirus disease 2019 (COVID-19) has emerged as a major public health challenge worldwide. A comprehensive understanding of clinical characteristics and immune responses in asymptomatic carriers and symptomatic patients with COVID-19 is of great significance to the countermeasures of patients with COVID-19. Herein, we described the clinical information and laboratory findings of 43 individuals from Hunan Province, China, including 13 asymptomatic carriers and 10 symptomatic patients with COVID-19, as well as 20 healthy controls in the period from 25 January to 18 May 2020. The serum samples of these individuals were analyzed to measure the cytokine responses, receptor-binding domain (RBD), and nucleocapsid (N) protein-specific antibody titers, as well as SARS-CoV-2 neutralizing antibodies (nAbs). For cytokines, significantly higher Th1 cytokines including IL-2, IL-8, IL-12p70, IFN-γ, and TNF-α, as well as Th2 cytokines including IL-10 and IL-13 were observed in symptomatic patients compared with asymptomatic carriers. Compared with symptomatic patients, higher N-specific IgG4/IgG1 ratio and RBD-specific/N-specific IgG1 ratio were observed in asymptomatic carriers. Comparable nAbs were detected in both asymptomatic carriers and symptomatic patients with COVID-19. In the symptomatic group, nAbs in patients with underlying diseases were weaker than those of patients without underlying diseases. Our retrospective study will enrich and verify the clinical characteristics and serology diversities in asymptomatic carriers and symptomatic patients with COVID-19.

## Introduction

Over the past 20 years, there have been two waves of betacoronavirus emerging, including severe acute respiratory syndrome CoV (SARS-CoV) in 2003 (Peiris et al., 2003) and Middle East respiratory syndrome CoV (MERS-CoV) in 2012 (Zaki et al., 2012). In December 2019, another betacoronavirus causing human pneumonia emerged and soon was isolated (Zhu et al., 2020). The etiologic agent was renamed severe acute respiratory syndrome coronavirus 2 (SARS-CoV-2), and the infection was named coronavirus disease 2019 (COVID-19) by the [World Health Organization WHO (WHO, 2020)]. As of 25 February 2022, SARS-CoV-2 had spread to 212 countries and regions, causing over 428 million infected cases and more than 5.91 million deaths across the globe (WHO, 2022a). The COVID-19 pandemic has seriously threatened public health safety and attacked the global economy.

Coronavirus disease 2019 is clinically characterized by fever, cough, acute respiratory distress syndrome (ARDS), and in some cases, death (Chen et al., 2020; Guan et al., 2020). Patients with COVID-19 present a broad spectrum of clinical presentation, including asymptomatic, mild, moderate, severe, and critical cases. According to a meta-analysis covering 29,776,306 individuals from January 2020 to February 2021, asymptomatic carriers account for 40.50% of all confirmed population (95% CI, 33.50–47.50%) (Ma et al., 2021). Besides, undergoing a mutate period from B.1.1.7 (Alpha), B.1.351 (Beta), P.1 (Gamma), and B.1.617.2 (Delta) to B.1.1.529 (Omicron), SARS-CoV-2 variants appeared to be more contagious and less pathogenicity, especially the currently global disseminated Omicron variant (Lewnard et al., 2022; WHO, 2022b). Compared with symptomatic patients with COVID-19, asymptomatic carriers exhibit a longer median length of viral shedding, weaker nAbs, and faster nAb decrease (Long et al., 2020). Moreover, transmission from asymptomatic carriers was estimated to account for more than half of all transmission, of which children and females are more likely to present as asymptomatic COVID-19 carriers (Johansson et al., 2021; Syangtan et al., 2021).

Some studies have attempted to elucidate the difference in immune response and other clinical characteristics between asymptomatic carriers and symptomatic patients with COVID-19 from different points of view (Liu et al., 2020, 2021; Long et al., 2020; Wang Y. et al., 2020; Wu et al., 2020; Zhou et al., 2020; Cai et al., 2021; Tutukina et al., 2021), including complete blood count, neutrophil-to-lymphocyte ratio, kidney function indicators, viral loads, and anti-RBD/anti-N antibody ratio, as well as other risk factors. Among them, an indicator of the neutralization potency of anti-RBD antibody quality has been established, and it revealed that high potency was a predictor of survival (Garcia-Beltran et al., 2021). Nevertheless, few consensuses have been achieved in terms of the intuitive and quantifiable indicators between asymptomatic carriers and symptomatic patients. Additional and specialized immunological analysis is required to better recognize the differences between these two groups.

In this study, we described the clinical characteristics and immune responses, including cytokine levels and SARS-CoV-2-specific antibodies, as well as nAbs in 13 asymptomatic carriers and 10 symptomatic patients with COVID-19. Longitudinal comparisons of immune response between asymptomatic carriers and symptomatic patients provide information and assist in the risk stratification and triage of patients with COVID-19, supporting the clinical diagnosing, prevention, and treatment of COVID-19.

## Materials and Methods

### Study Subjects

From 25 January to 18 May 2020, 23 individuals were enrolled and admitted to The Second Affiliated Hospital of Nanhua University (Hengyang, China), including 13 asymptomatic carriers and 10 symptomatic patients with COVID-19, and the serum samples were harvested on admission. Besides, 20 healthy volunteers from physical examination centers were involved, and the sera were collected as healthy controls in the same period. Clinical pathological data on patients with COVID-19 were retrieved from the electronic medical records. Individuals who tested positive for SARS-CoV-2 nucleic acids but did not exhibit symptoms were identified as asymptomatic carriers. Symptomatic patients were defined as those who tested positive for SARS-CoV-2 nucleic acids and accompanied by symptoms including fever, cough, fatigue, chest discomfort, sore throat, hyposmia, and rhinobyon. All the test results of SARS-CoV-2 nucleic acids were negative for the healthy human controls. The study protocol was approved by the Ethics Committee in the hospital, with informed consent waived for public health outbreak investigations.

### Laboratory and Chest Imaging Examination

Laboratory examination and chest CT imaging of 10 symptomatic patients with COVID-19 were involved in the previous study (Chung et al., 2020; Wang D. et al., 2020; Cai et al., 2021). The routine blood test was performed using Sysmex XN-3000, and detection panels include neutrophils, lymphocytes, monocytes, red blood cells (RBCs), hemoglobin, platelets (PLTs), and white blood cells (WBCs). The blood chemistry test was conducted with Cobas 8000, and liver function indexes include total bile acid (TBA), aspartate aminotransferase (AST), alanine aminotransferase (ALT), total bilirubin (TB), and direct bilirubin (DB); myocardial enzyme indexes include creatine kinase (CK), CK isoenzyme (CK-MB), and isozyme (MB); renal function indexes include blood urea nitrogen (BUN) and serum creatinine (SCr). Chest CT scans were performed by GE Discovery CT750 HD.

### Cytokine Analyses

Serum samples from the 13 asymptomatic carriers and 10 symptomatic patients with COVID-19 were involved to detect the cytokine levels by the Meso Scale Discovery (MSD) detection technology according to the manufacturer’s instruction as in the previous study (Cillo et al., 2021; Karaba et al., 2022). For the economy, 12 samples were randomly selected from 20 healthy controls for cytokine detection. Preparation of standard antigen is as follows: add 1 ml of diluent 2 to the standard antigen, shake and mix fully, place it at room temperature for 15–20 min, dilute it four times successively, and 7 standards and 1 blank sample should be prepared; preparation of antibody diluent is as follows: 60 μl of specific antibody was diluted to 3 ml with diluent 3; preparation of wash buffer is as follows: 1 × phosphate-buffered saline (PBS) (with 0.05% Tween-20); preparation of plate reading buffer is as follows: configure 2 × plate reading buffer; after three washes of the MSD plate with 150 μl wash buffer, 50 μl sample or standard antigen was added to each well, followed by incubation at room temperature for 2 h. After three washes, 25 μl detection antibodies were added and incubated at room temperature for 2 h. Finally, 150 μl of the plate reading buffer was added to each well. Data were acquired on the MESO QuickPlex SQ 120. Sample concentrations for each marker were then calculated based on the respective standard curve.

### SARS-CoV-2 Specific Antibody Detection

The SARS-CoV-2 specific antibody level of 13 asymptomatic carriers and 10 symptomatic patients with COVID-19 were detected. Binding antibodies against the SARS-CoV-2 RBD and N protein were detected using an enzyme-linked immunosorbent assay (ELISA).

#### Antibody Subtype Detection

Serum samples were subjected to an ELISA for N and RBD-specific IgE, IgM, IgG, IgG1, IgG2a, IgG3, and IgG4 antibody detection as in the previous study (Jiang et al., 2021; Yan et al., 2022). First, 96-well microtiter plates (Corning-Costar, Corning, NY, United States) were coated overnight at 4°C with recombinant SARS-CoV-2 N or RBD protein from SARS-CoV-2 Wuhan-Hu-1 strain (NCBI accession no. NC_045512.2) using baculovirus-insect cells (Sino Biological, Beijing, China) at 1 μg/ml. Following three PBST washes and blocking for 2 h at 37°C with PBS containing 3% bovine serum albumin (BSA), the plates were incubated with 1:200 dilutions of samples in PBS containing 0.5% (w/v) BSA at 37°C for 1 h. After another three washes with PBST, the plates were incubated at 37°C for 1 h with the following HRP-labeled goat antibodies: anti-human IgM (1:2,000; Southern Biotech, Birmingham, AL, United States) and anti-human IgG (1:5,000; Bioworld Technology, Inc., St. Louis Park, MN, United States); HRP-labeled mouse antibodies: anti-human IgE (1:5,000; Southern Biotech, Birmingham, AL, United States), anti-human IgG1 (1:5,000; Southern Biotech), anti-human IgG2 (1:5,000; Southern Biotech), anti-human IgG3 (1:5,000; Southern Biotech), and anti-human IgG4 (1:5,000; Southern Biotech). After the final three washes, 100 μl tetramethylbenzidine (TMB) substrate was added to each well, and the color development was stopped with 50 μl/well H_2_SO_4_ for plate reading at 450 nm (Bio-Rad, Hercules, CA, United States).

#### IgG Detection

Notably, 96-well microtiter plates (Corning-Costar, Corning, NY, United States) were coated overnight at 4°C with recombinant RBD protein at 1 μg/ml. Following three PBST washes and blocking for 2 h at 37°C with PBS containing 3% BSA, the plates were incubated with 1:80-1:163,840 dilutions of samples in PBS containing 0.5% (w/v) BSA at 37°C for 1 h. After another three PBST washes, the plates were incubated at 37°C for 1 h with the following HRP-labeled goat anti-human IgG antibodies (1:5,000; Bioworld Technology, Inc., St. Louis Park, MN, United States). After the final three washes, 100 μl TMB substrate was added to each well, and the color development was stopped with 50 μl/well H_2_SO_4_ for plate reading at 450 nm (Bio-Rad, Hercules, CA, United States).

### Neutralizing Antibody Detection

The nAb test of 10 symptomatic patients with COVID-19, 13 asymptomatic carriers, and 20 healthy controls was detected following the method of our previous study (Yan et al., 2022). All sera were heat-inactivated at 56°C for 30 min, then diluted in 96-well plates with 2-fold serial dilutions (from 1:20 to 1:40,960), mixed with 100 TCID_50_ of SARS-CoV-2 (Beta-Cov/Wuhan/AMMS01/2020), and incubated at 37°C, 5% CO_2_ for 1 h. Vero E6 cells were mixed with the virus-serum mixture in a volume of 50 μl/well. The virus mixture without serum and blank cells served as the control. Plates were incubated at 37°C, 5% CO_2_ for 48 h. The nAb titer of each sample was the reciprocal of the serum dilution that protected cells from cytopathic effect (CPE).

### Statistical Analyses

GraphPad Prism 8.0 software (GraphPad Software Inc., San Diego, CA, United States) was used to analyze the data, which are expressed as the mean ± standard error of the mean (SEM). Significant differences between groups were determined using one-way ANOVA. *P* < 0.05 was considered statistically significant.

## Results

### Basic Information and Clinical Manifestation

**FIGURE 1:**
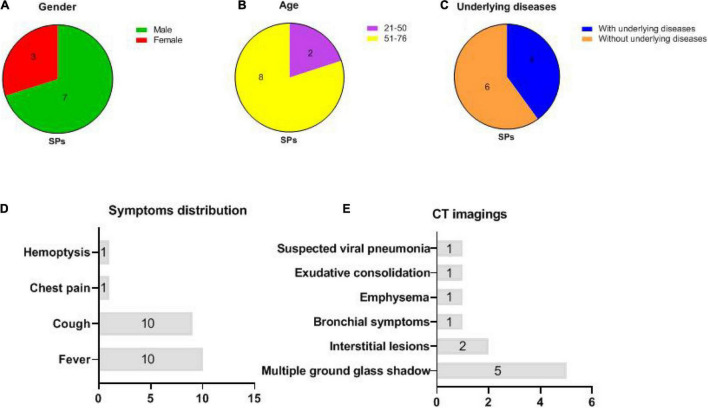
Basic information and clinical manifestations of 10 symptomatic patients (SPs) (same abbreviations in subsequent figures). **(A)** Gender distributions of SPs: 3 women and 7 men. **(B)** Age distributions of SPs: 8 individuals were between 51 and 76 while 2 individuals were between 21 and 50 years old. **(C)** 4 SPs with underlying diseases, whereas 6 SPs without underlying disease were recorded. **(D)** Distributions of clinical symptoms in SPs: fever (10/10) and cough (10/10) were the most frequently recorded. **(E)** CT imaging: multiple ground glass shadows were seen in 50% (5/10) individuals; interstitial lesions, bronchial symptoms, emphysema, exudative consolidation, and suspected viral pneumonia were recorded.

**FIGURE 2:**
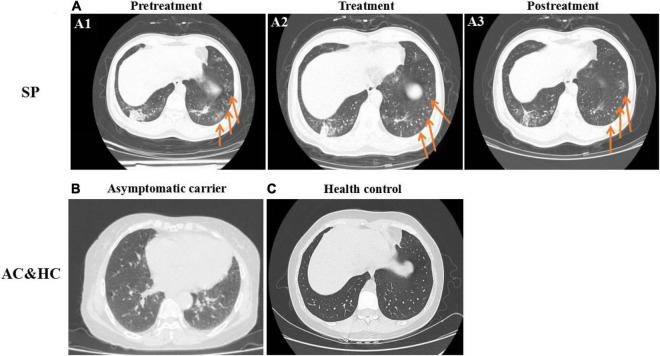
Typical chest CT imaging in SPs, asymptomatic carriers (ACs), and healthy controls (HCs) (same abbreviations in subsequent figures). **(A)** CT imaging in SPs. **(A1)** Pretreatment. **(A2)** Treatment. **(A3)** Posttreatment. **(B)** CT imaging in ACs. **(C)** CT imaging in HCs. Bilateral pneumonia and multiple ground glass shadows were obvious in SPs and were marked with arrows **(A1–A3)**, whereas pulmonary lesions in ACs **(B)** were not obvious compared with the control **(C)**.

The gender, age, underlying diseases, clinical symptoms, and computed tomography (CT) imaging information of 10 symptomatic patients were summarized in Figure 1. In symptomatic patients, 70% (7/10) men and 30% (3/10) women were included. Elderly people (51–76 years old) account for 80% (8/10) of all symptomatic patients, while young people (21–50 years old) account for 20% (2/10), with a median age of 57.6 years (21–76 years old). Underlying diseases were recorded in 60% (6/10) individuals in symptomatic patients, including hypertension, chronic bronchitis, type 2 diabetes, and coronary disease. Patients with COVID-19 exhibited manifestations of viral pneumonia including fever (seen in 100% of patients), cough (seen in 100% of patients), and chest discomfort. Multiple ground glass shadows and interstitial lesions were frequently observed in lung lesions by CT. Typical CT imaging of symptomatic patients and asymptomatic carriers with COVID-19 is shown in Figure 2. The above results indicated that men and elderly people with underlying diseases are the main risk factors for patients with COVID-19; among them, cough, fever, and multiple ground glass shadows were predominant clinical characteristics of symptomatic patients with COVID-19.

### Body Temperature and Hematology Parameters of Symptomatic Patients With COVID-19

Body temperature and hematology parameters of 10 symptomatic patients with COVID-19 were recorded. As shown in Supplementary Figure 1, a rise in body temperature was observed in 90% (9/10) of patients on admission. The temperature of all patients fluctuated and returned to the normal range after 7 days following hospitalization (Supplementary Figure 1A). On admission, 60% (6/10) of patients exhibited neutrophils above the normal range, which returned to normal after treatment (Supplementary Figure 1B). Lymphocytopenia was observed in 80% (8/10) of patients and returned to normal posttreatment (Supplementary Figure 1C). The patients with mononuclear cells above the normal range account for 20% (2/10) before treatment. During the treatment period, the monocytes of 30% (3/10) patients showed large fluctuations, but in the end, the monocytes of all patients returned to their normal range (Supplementary Figure 1D). The RBCs, hemoglobin, PLTs, and WBCs were relatively stable during the disease course (Supplementary Figures 1E–H). The above results indicated that increased neutrophil counts and lymphocytopenia are the most frequently observed parameters in symptomatic patients with COVID-19, accompanied by increased mononuclear cells.

### Blood Biochemical of Symptomatic Patients With COVID-19

Blood biochemical indicators of 10 symptomatic patients with COVID-19 are summarized in Supplementary Figure 2. For liver function (Supplementary Figure 2A), elevated TBA, AST, ALT, and DB levels were observed in 10% (1/10), 10% (1/10), 20% (2/10), and 20% (2/10) patients, respectively (Supplementary Figure 2A). Abnormal TB was not observed. For renal function (Supplementary Figure 2B), Cre and BUN of all individuals were in the normal range. For heart function (Supplementary Figure 2C), 20% (2/10) of patients exhibited elevated MB. CK and CK-MB were in the normal range in all symptomatic patients. Consequently, elevated liver function indicators such as ALT and DB levels are more obvious in symptomatic patients with COVID-19.

### Cytokine Measurement

**FIGURE 3:**
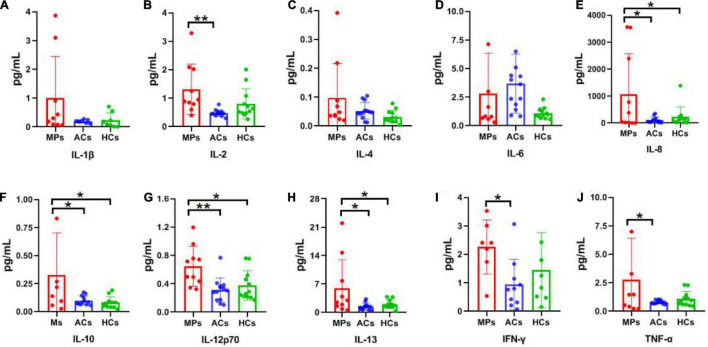
Cytokine detection results of SPs, ACs, and HCs. Red, blue, and green columns refer to SPs (10 individuals), ACs (13 individuals), and HCs (12 individuals), respectively. Unit: pg/ml. **(A)** IL-1β. **(B)** IL-2. **(C)** IL-4. **(D)** IL-6. **(E)** IL-8. **(F)** IL-10. **(G)** IL-12p70. **(H)** IL-13. **(I)** IFN-γ. **(J)** TNF-α. Data are presented as means ± SEM. *P < 0.05, **P < 0.01.

Cytokine detection results of interleukin-1β (IL-1β), IL-2, IL-4, IL-6, IL-8, IL-10, IL-12p70, IL-13, interferon-gamma (IFN-γ), and tumor necrosis factor-alpha (TNF-α) in asymptomatic carriers, symptomatic patients, and healthy controls are summarized in Figure 3. Of which IL-1β, IL-2, IL-8, IL-12p70, IFN-γ, and TNF-α are Th1 cytokines, while IL-4, IL-6, IL-10, IL-13 are Th2 cytokines. For Th1 cytokines, significantly higher IL-2, IL-12p70 (*P* < 0.01) as well as IL-8, IFN-γ, and TNF-α (*P* < 0.05) levels were observed in symptomatic patients compared with asymptomatic carriers (Figures 3B,E,G,I,J). For Th2 cytokines, significantly higher IL-10 and IL-13 were observed in symptomatic patients compared with asymptomatic carriers (*P* < 0.05) (Figures 3F,H). No obvious difference was observed among the above-mentioned three groups regarding IL-1β, IL-4, and IL-6 levels (Figures 3A,C,D). The above results showed that the cytokine levels are much higher in symptomatic patients than those in asymptomatic carriers, relating to clinical symptoms.

### Antibody Responses

**FIGURE 4:**
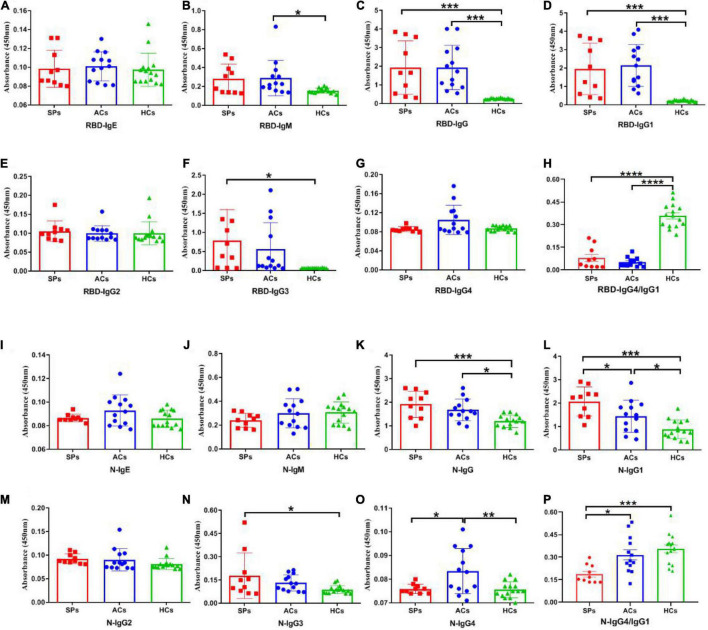
SARS-CoV-2 RBD and N-specific binding antibodies in SPs, ACs, and HCs. IgE, IgM, IgG, IgG1, IgG2, IgG3, and IgG4 antibody RBD protein of SARS-CoV-2 were detected in sera **(A–G)**. IgE, IgM, IgG, IgG1, IgG2, IgG3, and IgG4 antibody against N protein of SARS-CoV-2 were detected in sera **(I–O)**. The ratio of IgG4/IgG1 against RBD and N protein of SARS-CoV-2 in SPs and ACs were summarized **(H,P)**. Data are presented as means ± SEM. *P < 0.05, **P < 0.01,***P < 0.001, ****P < 0.0001.

To better understand the antibody responses in serum from symptomatic patients and asymptomatic carriers, the IgE, IgM, IgG, IgG1, IgG2, IgG3, and IgG4 antibody responses against the N and RBD proteins of SARS-CoV-2 were detected by ELISA (Figure 4). No significant differences in IgE antibody levels were observed among symptomatic patients, asymptomatic carriers, and healthy controls (Figures 4A,I). The RBD-targeting IgM antibody in symptomatic patients was significantly higher than that in asymptomatic carriers (*P* < 0.05) (Figure 4B). Significantly higher RBD-targeting IgG titers were observed in both symptomatic and asymptomatic groups compared with the healthy controls (*P* < 0.001), whereas more obvious increased N-targeting IgG was exhibited in the symptomatic group (*P* < 0.001) than that of the asymptomatic group (*P* < 0.05) (Figures 4C,K). Antibody subtype analysis indicated that virus-specific antibody was IgG1-biased, and IgG1 response was corresponded with IgG (Figures 4C,D,K,L). IgG3 in the symptomatic group was significantly higher than in healthy controls (*P* < 0.05) (Figures 4F,N). No obvious difference in IgG2 was observed (Figures 4E,M). Of interest, N-targeting IgG4 antibodies were more obvious in asymptomatic carriers (*P* < 0.05) (Figure 4O). Furthermore, the ratio of RBD and N-targeting IgG4/IgG1 was compared. The results indicated that the ratio of RBD-targeting IgG4/IgG1 was comparable between asymptomatic carriers and symptomatic patients with COVID-19 while a significantly lower N-targeting IgG4/IgG1 ratio was observed in symptomatic patients than that of asymptomatic carriers (Figures 4H,P). The above results suggested that the ratio of N-targeting IgG4/IgG1 may be served as a potential indicator of symptomatic patients.

**FIGURE 5:**
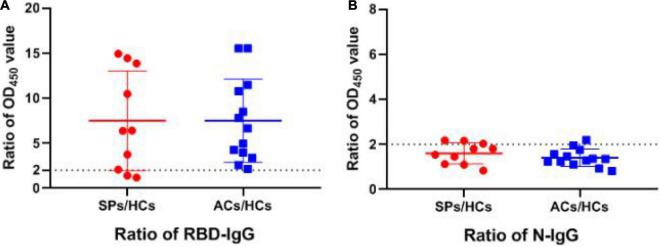
The ratio of OD_450_ value of RBD and N-specific IgG between SPs and ACs and HCs. **(A)** The ratio of RBD-specific IgG OD_450_ value between experiment group and HCs. **(B)** The ratio of N-specific IgG OD_450_ value between experiment group and HCs. Data are presented as means ± SEM.

**FIGURE 6:**
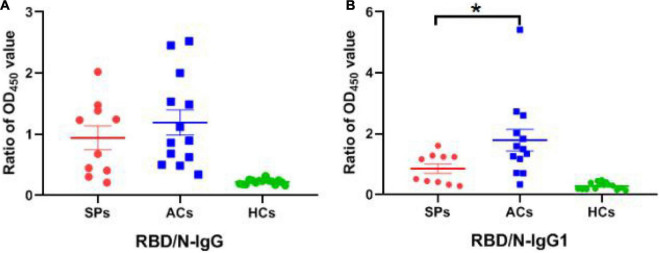
The ratio of OD_450_ value between RBD-specific and N-specific IgG and IgG1 in SPs, ACs, and HCs. **(A)** The ratio of OD_450_ value between RBD-specific IgG and N-specific IgG. **(B)** The ratio of OD_450_ value between RBD-specific IgG1 and N-specific IgG1. *P < 0.05.

Subsequently, the positive rate of IgG was compared between these groups. The OD_450_ of the serum ≥ cut-off was determined as positive. For RBD-targeting IgG, an 80% (8/10) positive rate was seen in symptomatic patients with COVID-19, and a 100% (13/13) positive rate was seen in asymptomatic carriers (Figure 5A). For N-targeting IgG, a 30% (3/10) positive rate was seen in symptomatic patients with COVID-19, and a 7.69% (1/13) positive rate was seen in asymptomatic carriers (Figure 5B). The above results indicated that RBD-specific IgG was more sensitive than that N-specific IgG antibody response in asymptomatic carriers. Furthermore, the ratio of RBD-specific/N-specific IgG and IgG1 was compared (Figures 6A,B). A trend of higher RBD-specific/N-specific IgG ratio was observed while the RBD-specific/N-specific IgG1 ratio was significantly higher in asymptomatic carriers compared with symptomatic patients (*P* < 0.05). The above results exhibited that the ratio of RBD-specific/N-specific IgG1 was a more sensitive indicator than RBD-specific/N-specific IgG.

**FIGURE 7:**
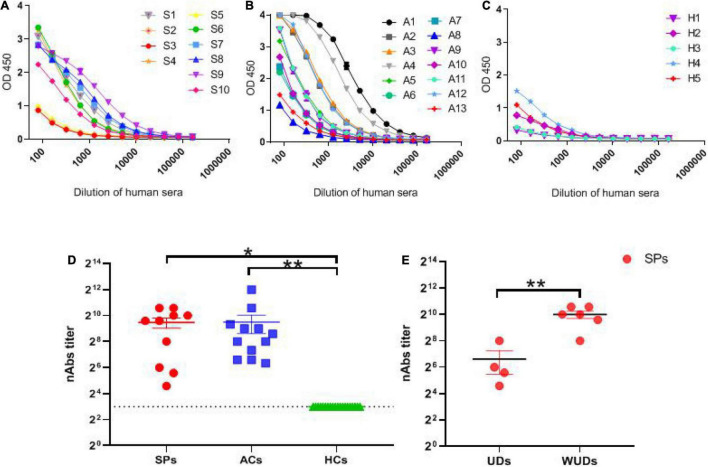
SARS-CoV-2-specific IgG and nAb response in SPs, ACs, and HCs. **(A–C)** 1 μg/ml RBD coating, OD_450_ values with the dilution of human sera. **(A)** SPs. **(B)** ACs. **(C)** HCs. **(D)** Comparison of the level of nAbs in SPs, ACs, and HCs. **(E)** Comparison of the level of nAbs between patients with underlying diseases (UDs) and patients without underlying diseases (WUDs) in both SPs. Data are presented as means ± SEM. *P < 0.05, **P < 0.01.

As shown in Figures 7A–C, with the increase in serum dilution, the optical density value of asymptomatic carriers decreased more significantly compared with the symptomatic group, indicating that symptomatic patients acquired a stronger binding to SARS-CoV-2 RBD protein than asymptomatic carriers.

The nAb titers of the sera from the samples were detected with live SARS-CoV-2. The nAb titers could be detected in symptomatic patients and asymptomatic carriers, and no significant difference was observed between the two groups (Figure 7D). Furthermore, symptomatic patients with underlying diseases tended to produce significantly lower nAbs compared with their counterparts (*P* < 0.05) (Figure 7E). Overall, these results showed that potent human nAbs could be elicited by SARS-CoV-2 infection, and the nAb titers produced by patients with underlying diseases are much lower compared with their counterparts.

## Discussion

With the continuous efforts of scientists worldwide, people have got a better grasp of the clinical symptoms and hematological signs of symptomatic patients with COVID-19 (Terpos et al., 2020; WHO, 2020; Wu et al., 2020; Zhou et al., 2020; Zhu et al., 2020). Our study confirmed and presented several aspects of COVID-19 clinical manifestations. Fever, cough, elevated neutrophils, lymphopenia, erythrocytosis, and multiple ground-glass opacity of lungs are the most frequently observed clinical characteristics of symptomatic patients with COVID-19. Meanwhile, serum liver function index (ALT and DB) abnormalities are common in symptomatic patients with COVID-19. In addition to the above, the neutrophil-to-lymphocyte (NLR) ratio >6.11 (Cai et al., 2021), elevated BUN (Liu et al., 2020), decreased blood uric acid level, D-dimer concentrations >1 μg/L, a greater sequential organ failure assessment (SOFA) score, high-sensitivity cardiac troponin I, and lactate dehydrogenase were correlated with increased risk of in-hospital death (Zhou et al., 2020). Interestingly, a scoring model has been developed to accurately and dynamically determine the death risk of hospitalized patients with COVID-19 based on blood routine examination indicators, namely, the PAWNN score (Liu et al., 2021). Using the Cox proportional hazard regression model, five risk factors were involved to construct the PAWNN score, including PLT counts, age, WBC counts, neutrophil counts, and neutrophil/lymphocyte ratio. The above-mentioned clinical manifestations and hematology information enrich our knowledge of COVID-19 and support auxiliary methods for the clinical diagnosis and prognosis of COVID-19.

“Cytokine Storm,” a systemic hyper-inflammation that can cause rapid clinical deterioration and fatality, illustrates the immune system’s inability to eradicate SARS-CoV-2, which contributes to the development of ARDS and multiple organ failure in COVID-19 cases (McGonagle et al., 2020; Merad and Martin, 2020; Ye et al., 2020). The dynamics of serum cytokine levels of patients with severe COVID-19 have been in-depth elucidated. IL-6, IL-8, IL-10, and TNF-α were increased in severe cases of COVID-19 while IFN-α, IL-1β, IL-4, and IL-15 were enriched in mild cases (Del Valle et al., 2020; Huang et al., 2020; Ruan et al., 2020; Garcia-Beltran et al., 2021). IL-2 and IL-7 were enriched in both severe and mild cases. The excessive inflammatory response characterized by the upregulated IL-6 and TNF-α is a typical immune disorder in patients with severe COVID-19 (Hadjadj et al., 2020). Our study focuses on cytokine diversity between symptomatic patients and asymptomatic carriers. Interestingly, obviously elevated IL-2, IL-8, IL-10, TNF-α, and IL-12p70 were observed in symptomatic patients with COVID-19 compared with asymptomatic carriers, which were consistent with the results in that of patients with COVID-19.

Antibodies are key indicators following SARS-CoV-2 infection. Several studies have attempted to elucidate antibody response in relation to COVID-19 severity. Severe patients are biased toward lower-than-predicted neutralization titers, suggesting that they harbor anti-RBD IgG antibodies that did not contribute to neutralization (Garcia-Beltran et al., 2021). Asymptomatic individuals are not equivalent to weaker immune responses. As has been described (Tutukina et al., 2021), a higher anti-RBD/anti-N IgG ratio reflects less severe symptoms. A higher anti-RBD/anti-N IgG ratio in asymptomatic carriers compared with symptomatic patients was confirmed in this study. Furthermore, we suggested that the higher anti-RBD/anti-N IgG1 ratio was more obvious in asymptomatic carriers. Particularly, in terms of higher IgG4 antibodies in asymptomatic carriers, we proposed for the first time that the higher N-specific IgG4/IgG1 ratio may be another sensitive indicator in asymptomatic carriers. The above sensitive indicators demand further larger-scale clinical investigations. Previous study showed that the asymptomatic group exhibited a more obvious decrease in nAbs levels compared with the symptomatic group. From the shedding of antigen to the level of antibody, subsequently the duration of antibody, the results varied among different research groups (Long et al., 2020; Wang Y. et al., 2020). We did not observe differences in the level of nAbs between asymptomatic carriers and symptomatic patients. Although the protective immunity of COVID-19 has yet been clearly elucidated, nAbs are thought to play a key role in the control of SARS-CoV-2 infection (Mercado et al., 2020; Yu et al., 2020; Dispinseri et al., 2021), which explained the relatively consistent but potent nAbs in both groups in this study.

This study has some limitations. It is a short-term case analysis in a specific district, so the samples size is relatively small. Besides, the dynamic characteristics of the immune response during SARS-CoV-2 infection were not assessed. Once the kinetics of virus shedding and changes in antibody titers during the progression of COVID-19 was obtained, a better grasp of the delicate difference between symptomatic patients and asymptomatic carriers with COVID-19 will be obtained.

**FIGURE 8:**
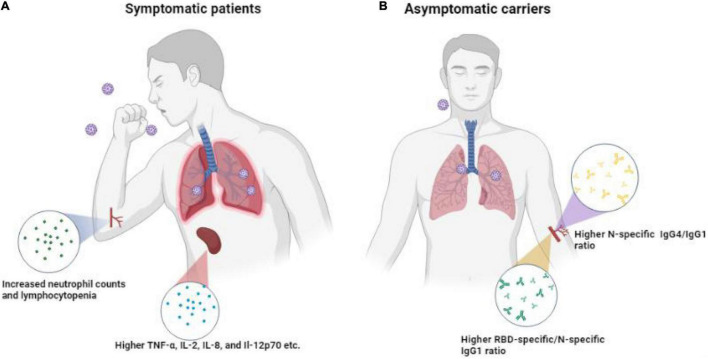
Differences in immune response in SPs, ACs, and HCs. **(A)** Symptomatic patients exhibited higher anti-RBD/anti-N antibody ratios and higher TNF-α, IL-2, IL-8, and IL-12p70. **(B)** Asymptomatic carriers exhibited higher N-specific IgG4/IgG1 ratio antibodies.

To sum up, differences in immune response between asymptomatic carriers and symptomatic patients with COVID-19 are depicted in Figure 8. Asymptomatic carriers exhibited higher N-specific IgG4/IgG1 ratio and higher RBD-specific/N-specific IgG1 while symptomatic patients exhibited increased neutrophil counts and lymphocytopenia as well as higher TNF-α, IL-2, IL-8, and IL-12p70. Collectively, our study of clinical and serological manifestations revealed distinct immunity patterns of SARS-CoV-2 infection in symptomatic and asymptomatic patients. Our findings contribute to the understanding of the interaction of SARS-CoV-2 and host immune system, improving the risk stratification and management of patients with COVID-19.

## Data Availability Statement

The raw data supporting the conclusions of this article will be made available by the authors, without undue reservation.

## Ethics Statement

The studies involving human participants were reviewed and approved by the Medical Ethics Committee of The Second Affiliated Hospital of Nanhua University. The patients/participants provided their written informed consent to participate in this study. Written informed consent was obtained from the individual(s) for the publication of any potentially identifiable images or data included in this article.

## Author Contributions

YG and BL: conceptualization. FY: methodology and project administration. EL: software. EL and SW: formal analysis and writing original draft preparation. WH: investigation. JH: resources. LL: data curation. XZ: writing review and editing. XX and SY: supervision. BL: funding acquisition. All authors have read and agreed to the published version of the manuscript.

## Conflict of Interest

The authors declare that the research was conducted in the absence of any commercial or financial relationships that could be construed as a potential conflict of interest.

## Publisher’s Note

All claims expressed in this article are solely those of the authors and do not necessarily represent those of their affiliated organizations, or those of the publisher, the editors and the reviewers. Any product that may be evaluated in this article, or claim that may be made by its manufacturer, is not guaranteed or endorsed by the publisher.
