# Supplementary material for: Clinical Characteristics of Immune Response in Asymptomatic Carriers and Symptomatic Patients With COVID-19
Source: Front Microbiol. 2022 May 24;13:896965. doi: 10.3389/fmicb.2022.896965 (PMC9171238; doi:10.3389/fmicb.2022.896965)
Supplement: Supplementary file 1 [file Data_Sheet_1.docx]

**Supplement Table 1.** Clinical presentations of 10 symptomatic individuals with COVID-19.


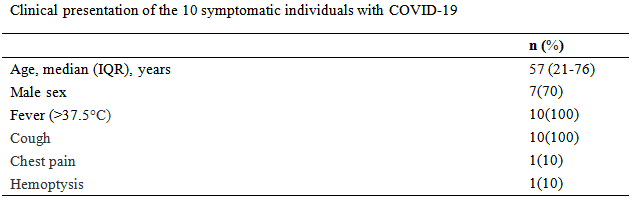


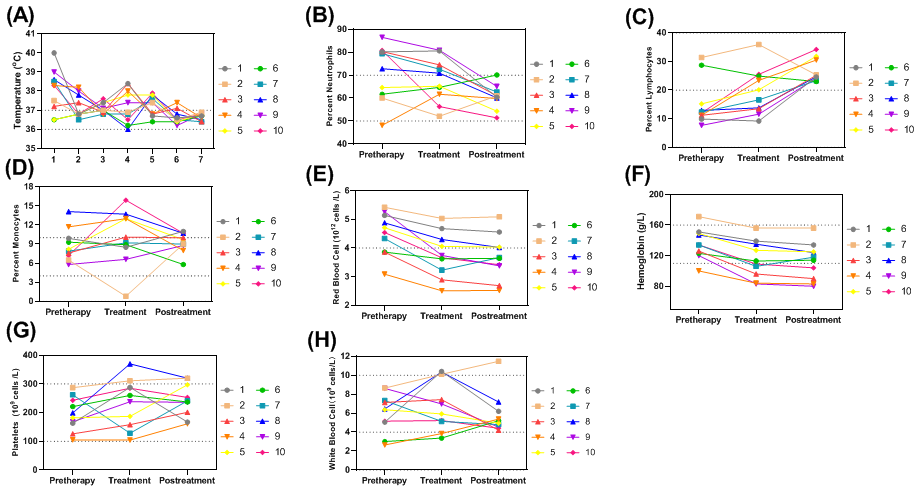


**Supplement Figure 1.** Body temperature and hematology parameters of 10 SPs in the period of pretreatment, treatment and post-treatment. A: Body temperature. B: Neutrophils. C: Lymphocytes. D: Monocytes. E: Red blood cells. F: Hemoglobin. G: Platelets. H: White blood cells.


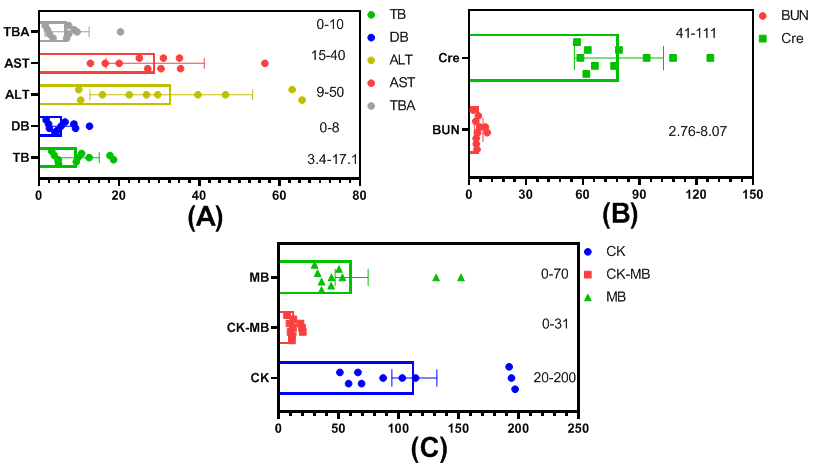


**Supplement Figure 2.** Blood biochemistry results in 10 SPs. A: Liver function indexes including TBA, AST, ALT, DB and TB; B: Renal function indexes including BUN, and Cre; C: Myocardial enzyme indexes including CK, CK-MB, and MB.
